# Differential contributory roles of nucleotide excision and homologous recombination repair for enhancing cisplatin sensitivity in human ovarian cancer cells

**DOI:** 10.1186/1476-4598-10-24

**Published:** 2011-03-08

**Authors:** Qi-En Wang, Keisha Milum, Chunhua Han, Yi-Wen Huang, Gulzar Wani, Jürgen Thomale, Altaf A Wani

**Affiliations:** 1 Department of Radiology, The Ohio State University, Columbus, OH 43210, USA; 2 Comprehensive Cancer Center, The Ohio State University, Columbus, OH 43210, USA; 3 Institut für Zellbiolgoie, Universitätsklinikum Essen, Germany; 4 Department of Molecular and Cellular Biochemistry, The Ohio State University, Columbus, OH 43210, USA; 5 DNA Research Chair, King Saud University, Riyadh, Saudi Arabia

## Abstract

**Background:**

While platinum-based chemotherapeutic agents are widely used to treat various solid tumors, the acquired platinum resistance is a major impediment in their successful treatment. Since enhanced DNA repair capacity is a major factor in conferring cisplatin resistance, targeting of DNA repair pathways is an effective stratagem for overcoming cisplatin resistance. This study was designed to delineate the role of nucleotide excision repair (NER), the principal mechanism for the removal of cisplatin-induced DNA intrastrand crosslinks, in cisplatin resistance and reveal the impact of DNA repair interference on cisplatin sensitivity in human ovarian cancer cells.

**Results:**

We assessed the inherent NER efficiency of multiple matched pairs of cisplatin-sensitive and -resistant ovarian cancer cell lines and their expression of NER-related factors at mRNA and protein levels. Our results showed that only the cisplatin-resistant ovarian cancer cell line PEO4 possessed an increased NER capacity compared to its inherently NER-inefficient parental line PEO1. Several other cisplatin-resistant cell lines, including CP70, CDDP and 2008C13, exhibited a normal and parental cell-comparable NER capacity for removing cisplatin-induced DNA intrastrand cross-links (Pt-GG). Concomitant gene expression analysis revealed discordance in mRNA and protein levels of NER factors in various ovarian cancer cell lines and NER proteins level were unrelated to the cisplatin sensitivity of these cell lines. Although knockdown of NER factors was able to compromise the NER efficiency, it only caused a minimal effect on cisplatin sensitivity. On the contrary, downregulation of BRCA2, a critical protein for homologous recombination repair (HRR), significantly enhanced the efficacy of cisplatin in killing ovarian cancer cell line PEO4.

**Conclusion:**

Our studies indicate that the level of NER factors in ovarian cancer cell lines is neither a determinant of their NER capacity nor of the sensitivity to cisplatin, and suggest that manipulation of the HRR but not the NER factor expression provides an effective strategy for sensitizing cisplatin-resistant tumors to platinating agents.

## Background

Since the introduction of inorganic platinum (Pt) drug molecule cisplatin into the clinic, platinum-based chemotherapy drugs have been in widespread use to treat various malignant tumors, including ovarian, testicular, head and neck, and lung cancers [1]. It is generally accepted that the anti-neoplastic activity of cisplatin results from its binding to DNA in target cells to induce DNA cross-links. Chemotherapy with cisplatin is initially effective for most patients. However, the majority eventually becomes refractory to platinum treatment and cisplatin resistance develops, which severely limits the effective use of platinum-based chemotherapeutic drugs.

Cisplatin forms primarily 1, 2-intrastrand cross-links between adjacent purines in DNA, e.g. *cis*-Pt(NH_3_)_2_d(GpG) (Pt-GG), with Pt bound to two adjacent guanines, and *cis*-Pt(NH_3_)_2_d(ApG) (Pt-AG), in which the Pt is bound to adenine and an adjacent guanine. These lesions contribute to 90% of total damage introduced by cisplatin. Other DNA damage introduced by cisplatin includes 1, 3-intrastrand cross-links (5-10%) and interstrand cross-links (1-2%) [2]. The cisplatin-induced intrastrand cross-links are mainly removed by nucleotide excision repair (NER). Thus, alteration of this DNA repair pathway is believed to confer resistance to platinum-based chemotherapy. The minor 1, 3-intrastrand cross-links are repaired more efficiently than 1, 2-intrastrand adducts, due to greater helical distortion introduced by this bulky adduct [3] and presumed shielding of 1, 2-intrastrand adducts from its binding to high-mobility group (HMG) proteins [4,5]. However, the repair of interstrand cross-links induced by cisplatin is more complex, and involves excision repair and homologous recombination (HR) [6].

In terms of lesion recognition, NER is the most versatile choice among all repair systems operational in living cells. This DNA repair system can eliminate a wide variety of helix-distorting lesions, e.g., UV-induced photolesions, Benzo[a]pyrene Diol Epoxide (BPDE) and cisplatin-induced bulky adducts. The complete NER reaction involves several biochemical steps including damage recognition, dual incision, and gap-filling DNA synthesis [7]. In human cells, the minimal set of NER components involved in performing repair reaction comprises XPA, XPC-hHR23B, XPG, RPA, ERCC1-XPF, TFIIH, PCNA, DNA polymerase δ or ε, and DNA ligase I [8]. It is becoming increasingly clear and acceptable that in mammalian cells, NER is mediated by the sequential assembly of repair proteins at the site of the DNA lesion [9-11]. HR is a conserved pathway for the repair of double-strand breaks (DSBs), with Rad51 recombinase playing a central role. BRCA2 is essential for efficient HR through conjunction with Rad51 [12]. BRCA2-deficient cancer cells are hypersensitive to DNA-crosslinking agents including cisplatin [13], as a consequence, women with BRCA1/2-mutated ovarian carcinoma have a better diagnosis than those without BRCA1/2- mutation if they receive platinum-based therapy [14].

Evidence for increased repair of platinum-induced DNA damage in resistant ovarian cancer cells has been demonstrated by many groups (see review [15]). However, most studies focused on the relationship between total DNA repair capacity and cisplatin resistance, as DNA repair efficiency was assessed by comparing total platinum-DNA adduct levels using atomic absorption spectrometry [16-18], or by examining unscheduled DNA synthesis (UDS)[19], or by determining reactivation of cisplatin-damaged plasmid DNA [18]. Since more than 90% of DNA damage induced by cisplatin is removed by NER, the relationship between NER and cisplatin resistance seems especially important. In addition, the results of correlation between cisplatin resistance with elevated levels of genes and proteins of the NER pathway are also contradictory [15]. In this study, we assessed the NER capacity of multiple pairs of cisplatin-sensitive and -resistant ovarian cancer cell lines and analyzed the expression of various NER factors at both mRNA and protein levels. Our data, indicating that high NER efficiency to remove cisplatin-induced DNA intrastrand cross-links does not always correlate to cisplatin resistance in ovarian cancer cell lines. Manipulation of HRR, but not NER factor expression, could enhance the sensitivity of cisplatin-resistant tumors to platinating agents, providing an important clinically relevant guidance about the potential manipulation of DNA repair pathways for sensitizing cisplatin-resistant tumors to platinating agents.

## Materials and methods

### Cell culture and treatment

The experiments were performed with three different groups of human ovarian cancer cell lines, each group having one cisplatin-sensitive parental cell line and one or two cisplatin-resistant variants (Table 1). The human ovarian cancer cell line A2780 and its resistant subline CP70 were kindly provided by Dr. Paul Modrich (Duke University). Another A2780-derivative resistant subline CDDP was kindly provided by Dr. Karuppaiyah Selvendiran and Dr. Periannan Kuppusamy (The Ohio State University). Ovarian cancer cell line 2008 and its resistant cell line 2008C13 were kindly provided by Dr. Francois X. Claret (University of Texas - M. D. Anderson Cancer Center). The A2780-derivative and 2008-derivative cisplatin-resistant cell lines were produced by intermittent, incremental exposure of the sensitive parental cell line to various concentrations of cisplatin. Cisplatin-sensitive ovarian cancer cell line PEO1 and -resistant PEO4, established from the same patient before treatment and after developing resistance to platinum-based chemotherapy, were kindly provided by Dr. Thomas C. Hamilton (Fox Chase Cancer Center). CP70 cells with overexpression of DDB2 (CP70-DDB2) were established in our lab [20]. All cell lines were maintained in RPMI 1640 supplemented with 10% fetal bovine serum, 100 μg/ml streptomycin and 100 units/ml penicillin. Cells were grown at 37°C in humidified atmosphere of 5% CO_2 _in air. Sensitivity to cisplatin in these ovarian cancer cell lines following one hour treatment was assessed by growth inhibition assay using 96-well plates as described later. The IC50 of each cell line was shown in Table 1.

**Table 1:** Cisplatin sensitivity and NER capacity of human ovarian cancer cell lines.

*Cell line*	***IC50 (μM)***^***1***^	***NER capacity to remove Pt-GG***^***2***^
A2780	2.97 ± 0.18	**++**
CP70	45.78 ± 0.10	**++**
CDDP	29.19 ± 9.43	**++**
2008	10.82 ± 0.16	**++**
2008C13	54.14 ± 0.82	**++**
PEO1	12.79 ± 1.15	-
PEO4	44.46 ± 4.76	**++**

^1^IC50 was determined after 1 h treatment with increasing concentrations of cisplatin. Cell survival was determined by methylene blue staining as described in the Methods.

^2^NER capacity was assessed by determining the removal rate of DNA lesions in cells following various culture times.

For cisplatin treatment, cells were maintained in medium with the desired doses of cisplatin (Sigma, St. Louis, MO) for 1 h, and then washed with PBS and followed by incubation in fresh cisplatin-free medium for varying times post-treatment. For UV exposure, the cultures were washed with PBS and irradiated with UV at 10 J/m^2 ^followed by incubation for varying times. UV-C light (254 nm) was delivered from a germicidal lamp at a dose rate of 0.5 J/m^2^/s, as measured by a UVX digital radiometer connected to a UVX-31 sensor (UVP, Inc., Upland, CA)

### Immunoslot-blot (ISB) analysis

Cells were pre-treated with hydroxyurea (HU) for 24 h to exclude cells from S phase [21], then UV irradiated or treated with cisplatin for 1 h, washed twice with PBS, and further cultured in HU containing medium for the desired time periods to ensure the inhibition of DNA replication [22]. The nuclei were isolated and treated with RNase for 1 h. The genomic DNA was then isolated with phenol/chloroform/isoamyl alcohol (25:24:1), precipitated with ethanol, and quantified using PicoGreen kit assay (Invitrogen, Carlsbad, CA). The same amounts of denatured DNA were applied to nitrocellulose membranes. Cisplatin-induced DNA intrastrand cross-links (Pt-GG) were detected with anti-Pt-GG antibody [23]. The intensity of each band was quantified, and the lesion concentrations were determined from a reference standards run in parallel to calculate the relative amounts of Pt-GG remaining at each time point.

### Host cell reactivation (HCR) assay

The HCR assay was performed to determine the DNA repair capacity of individual cell lines. For this study, the pCMV-Tag 2 expression control plasmid (containing the firefly luciferase gene, Stratagene, La Jolla, CA) was treated with cisplatin (1 or 10 μM) to introduce DNA damage into the plasmid DNA. Both the undamaged and the damaged pCMV-Tag 2 plasmid were then transfected into cells (0.5 μg/35-mm dish) using Lipofectamine 2000 transfection reagent (Invitrogen). As an internal control, the pGL4.73 plasmid (Promega, Madison, WI), which carries a renilla luciferase gene, was also co-transfected into the cells. The cells were harvested 2 days after transfection, and both firefly and renilla luciferase activities were determined from the transfected cells using a Dual Luciferase Activity Detection System (Promega). The activity of firefly luciferase in each experiment was calculated as relative activity to the renilla luciferase activity to minimize the experimental variations. The ratio of luciferase activities in the same cell line for both undamaged and damaged plasmid was used to determine the DNA repair capacity of the host cells.

### Western blotting analysis

Whole cell lysates were prepared by boiling cell pellets for 10 min in lysis buffer (2% SDS, 10% Glycerol, 62 mM Tris-HCl, pH 6.8 and a complete mini-protease inhibitor cocktail [Roche Applied Science]). After protein quantification with Bio-Rad D*c *Protein Assay (Bio-Rad Laboratories, Hercules, CA), equal amounts of proteins were loaded, separated on a polyacrylamide gel, and transferred to a nitrocellulose membrane. Protein bands were immuno-detected with appropriate antibodies, e.g., rabbit anti-XPC and anti-DDB2 antibodies generated in our laboratory [24], mouse anti-XPA, mouse anti-XPF, and mouse anti-Tubulin antibodies purchased from Santa Cruz Biotechnology Inc. (Santa Cruz, CA). Rabbit anti-XPG antibody purchased from Bethyl Laboratory (Montgomery, TX). Mouse anti-BRCA2 (Ab-1) antibody purchased from Calbiochem (Gibbstown, NJ).

### Real-time quantitative RT-PCR

Total RNA was purified from various cell samples using Trizol (Invitrogen). The cDNA was generated by reverse transcription using Superscriptase III (Invitrogen) and oligo (dT) in a 20 μl reaction containing 1 μg of total RNA. An aliquot of 0.5 μl cDNA was used in each 20 μl PCR reaction, using Applied Biosystem's Power SYBR Green PCR Master Mix and the reactions were run on an ABI 7500 Fast Real-Time PCR system. The following primers were used: XPA, forward, 5'- GCA GCC CCA AAG ATA ATT GA -3'; reverse, 5'- TGG CAA ATC AAA GTG GTT CA -3'; XPC, forward, 5'- GAC AAG CAG GAG AAG GCA AC -3'; reverse, 5'- GGT TCG GAA TCC TCA TCA GA -3'; XPF, forward, 5'- TGC GTG AAT TTC GAA GTG AG -3'; reverse, 5'- TGG AGA TGC ACT GGC TGT AG -3'; XPG, forward, 5'- GGG AAA CCT GAT CTC GAC AA -3'; reverse, 5'- TCA ATT CGG AGC TGT GTC TG -3'; ERCC1, forward, 5'- TTG TCC AGG TGG ATG TGA AA -3'; reverse, 5'- GCT GGT TTC TGC TCA TAG GC -3'; and GAPDH, forward, 5'- GAA GGT GAA GGT CGG AGT -3'; reverse, 5'- GAA GAT GGT GAT GGG ATT TC -3'.

### Cell survival measurement

Cells were seeded in 96-well plates at an initial density of 2 × 10^3^, incubated for 24 h, and treated with increasing doses of cisplatin for 1 h. All test concentrations were repeated in quadruplicates. After the drug treatment, cultures were incubated for another 72 h. At the end of the growth period, the cells were washed with PBS, fixed with 3.7% formaldehyde for 30 min, and stained with 1.0% methylene blue for 30 min. The plate was rinsed in running water and then left to dry. 100 μl solvent (10% acetic acid, 50% methanol and 40% H_2_O) was added to each well to dissolve the cells and, optical density (OD) of the released color was read at 660 nm. The relative cell survival was calculated with the values of mock-treated cells set as 100%.

### Transfection with siRNAs

siRNA SMARTpools designed to target human *XPA*, *XPF or XPG *were purchased from Dharmacon Inc (Denver, CO). siRNA directed against BRCA2 (5'- AAC AAC AAT TAC GAA CCA AAC -3') [25], and a scramble non-targeting siRNA, were synthesized by Dharmacon. 50 nM siControl, siXPF or siXPG were separately transfected into CP70 or CDDP cells using Lipofectamine 2000 transfection reagent (Invitrogen, Carlsbad, CA) according to the manufacture's instruction. 100 nM siControl, 50 nM siXPA + 50 nM siControl, 50 nM siBRCA2 + 50 nM siControl, and 50 nM siXPA + 50 nM siBRCA2 were transfected into PEO4 cells, respectively, using the same transfection procedure as described above.

### HR repair (HRR) measurement by immunofluorescence of γH2AX

PEO4 cells growing on the coverslips were transfected with various siRNA as described above for 48 h, irradiated at 10 Gy with RS-2000 X-ray Biological Irradiator, and further cultured for 1 or 24 h. Cells were fixed and permeabilized with 2% paraformaldehyde in 0.5% Triton X-100, and stained with mouse anti-γH2AX antibody, and anti-mouse IgG conjugated with Texas Red. Fluorescence images were obtained with a Nikon fluorescence microscope E80i (Nikon, Tokyo, Japan). The digital images were then captured with a cooled CCD camera and processed with the help of its SPOT software (Diagnostic Instruments, Sterling Heights, MI).

## Results

### Determination of cisplatin dose to induce equivalent Pt-GG in paired cisplatin-sensitive and -resistant ovarian cancer cell lines

**Figure 1:**
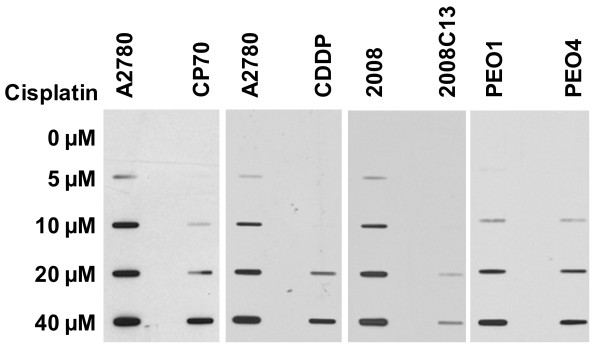
**Dose-response of cisplatin-induced Pt-GG in various cisplatin-sensitive and -resistant ovarian cancer cell lines**. A2780, CP70, CDDP, 2008, 2008C13, PEO1, and PEO4 cells were treated with cisplatin at various doses for 1 h, the total genomic DNA was isolated and the same amount of DNA was loaded for ISB. Cisplatin-induced intrastrand crosslinks were detected with anti-Pt-GG antibody.

One of the well known mechanisms of the cisplatin resistance is reduced drug intracellular uptake, which can result in lesser DNA damage and reduced cytotoxicity [26]. Thus, in order to study the changes of DNA repair capacity, it is essential to induce equivalent amounts of initial DNA lesions in different cell lines. To find the cisplatin doses that cause equivalent Pt-GG level in several pairs of cisplatin-sensitive and -resistant ovarian cancer cell lines A2780/CP70, A2780/CDDP, 2008/2008C13, and PEO1/PEO4, we quantitated the amount of Pt-GG in these cell lines after 1 h treatment with various doses of cisplatin. As shown in Figure 1, in comparison with the parental cisplatin-sensitive cancer cell lines A2780, 2008 and PEO1, their corresponding derivative resistant cell lines CP70, CDDP, 2008C13, and PEO4 exhibit a lower production of Pt-GG following the treatment with cisplatin at the same doses. For example, the amount of Pt-GG induced by 10 μM of cisplatin in A2780 cells is equivalent to that induced by 40 μM of cisplatin in CP70 and CDDP cells. The amount of Pt-GG produced by 7.5 μM of cisplatin in 2008 cells is equivalent to that produced by 40 μM of cisplatin in 2008C13 cells, and the amount of Pt-GG induced by 15 μM of cisplatin in PEO1 cells is equivalent to that induced by 20 μM of cisplatin in PEO4 cells. Therefore, in subsequent experiments, we used the different doses of cisplatin to treat different cell lines to ensure that the same initial amount of Pt-GG was produced when assessing the NER capacity in this study.

### NER efficiency of various cisplatin-sensitive and resistant ovarian cancer cell lines

**Figure 2:**
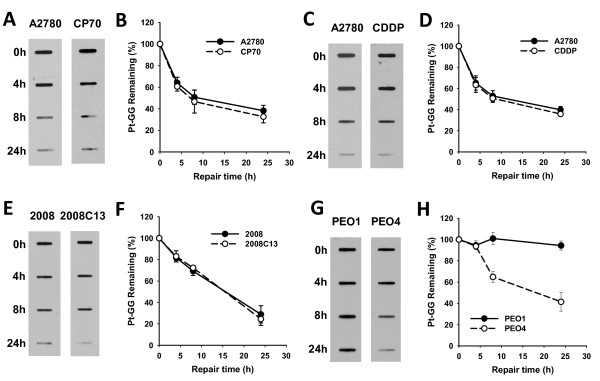
**NER efficiency of various cisplatin-sensitive and -resistant ovarian cancer cell lines determined by ISB**. A2780 and CP70 (A, B), A2780 and CDDP (C, D), 2008 and 2008C13 (E, F), PEO1 and PEO4 (G, H) cells were pre-treated with HU for 24 h, then treated with various concentration of cisplatin for 1 h (A2780: 10 μM; CP70: 40 μM; CDDP: 40 μM; 2008: 7.5 μM; 2008C13: 40 μM; PEO1: 15 μM; PEO4: 20 μM), and further cultured in HU containing medium for the indicated time periods. Total DNA was isolated and analyzed by ISB assay for cisplatin-induced intrastrand cross-links with anti-Pt-GG antibody. The intensity of each band was quantified by scanning images and processing with Alphaimager-2000 software. The relative percentage of remaining Pt-GG at different time points is an average of three independent repeats. (n = 3, Bar: SD).

**Figure 3:**
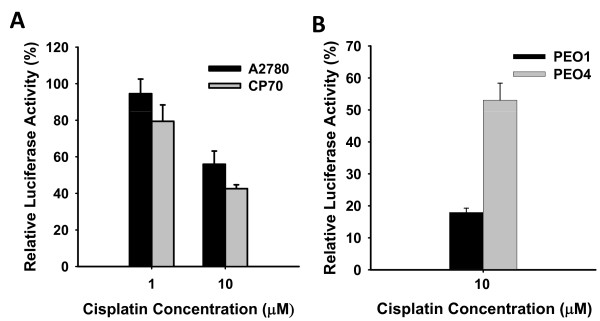
**DNA repair capacity of various cisplatin-sensitive and -resistant ovarian cancer cell lines determined by HCR**. A2780 and CP70 cells (A), PEO1 and PEO4 cells (B) were transfected with cisplatin-damaged and undamaged pCMV-Tag 2 plasmid. As an internal control, the pGL4.73 plasmid, which carries a renilla luciferase gene, was co-transfected with the pCMV-Tag 2 plasmid. The cells were harvested 2 days after transfection, both firefly and renilla luciferase activities were determined. The activity of firefly luciferase in each experiment was calculated as relative activity to the renilla luciferase activity to minimize the experimental variations. The ratio of luciferase activities in the same cell line for both undamaged and damaged plasmid was used to determine the DNA repair ability of the host cells. The relative luciferase activity is an average of three independent repeats. Bars represent standard deviation (SD).

It is believed that increased DNA repair efficiency is one of the reasons for the development of cisplatin resistance. To validate the contribution of NER pathway to the development of cisplatin resistance, we specifically detected and compared the removal rate of cisplatin-induced 1,2-intrastrand crosslinks (Pt-GG) between multiple matched pairs of cisplatin-sensitive and -resistant ovarian cancer cell lines by using immuno-slot blot assay with anti-Pt-GG antibody. Surprisingly, we did not observe any significant difference in the removal of Pt-GG between A2780 and CP70, A2780 and CDDP, as well as 2008 and 2008C13 cells (Figure 2A-F). On the other hand, however, the NER capacity of cisplatin-resistant PEO4 was significantly higher than that of cisplatin-sensitive PEO1 cells (Figure 2G &2H). We then assessed the NER capacity by an alternate assay based on host cell reactivation. As shown in Figure 3A &3B, when cisplatin-damaged pCMV-Tag 2 plasmids were transfected into various ovarian cancer cell lines for 48 h, the relative luciferase activities in A2780 and CP70 cell lines were comparable, while PEO4 cells exhibited significantly higher relative luciferase activity than PEO1 cells, indicating that A2780 and CP70 cell lines have similar DNA repair capacity in removing cisplatin-induced DNA lesions, whereas PEO4 cells have higher DNA repair capacity than PEO1 cells. In addition, ISB assay indicated that about 60% Pt-GG was removed after 24 h in PEO4 cells (Figure 2G &2H), a rate similar to that of other ovarian cancer cell lines, e.g., A2780, CP70, CDDP, 2008 and 2008C13 (Figure 2A-F), indicating that this cell line has normal NER capacity. However, severe impairment of NER capacity of PEO1 cells is evident by the lack of Pt-GG removal even at 24 h post-treatment (Figure 2G &2H). These data indicate that NER capacity is not increased in the acquired cisplatin-resistant ovarian cancer cell lines derived from cancer cells that already have high NER efficiency. In contrast, if the cisplatin-sensitive cells have a deficient NER, its derivative resistant cells may display enhanced NER efficiency (Table 1).

### Expression of NER factors in various cisplatin-sensitive and -resistant ovarian cancer cell lines

**Figure 4:**
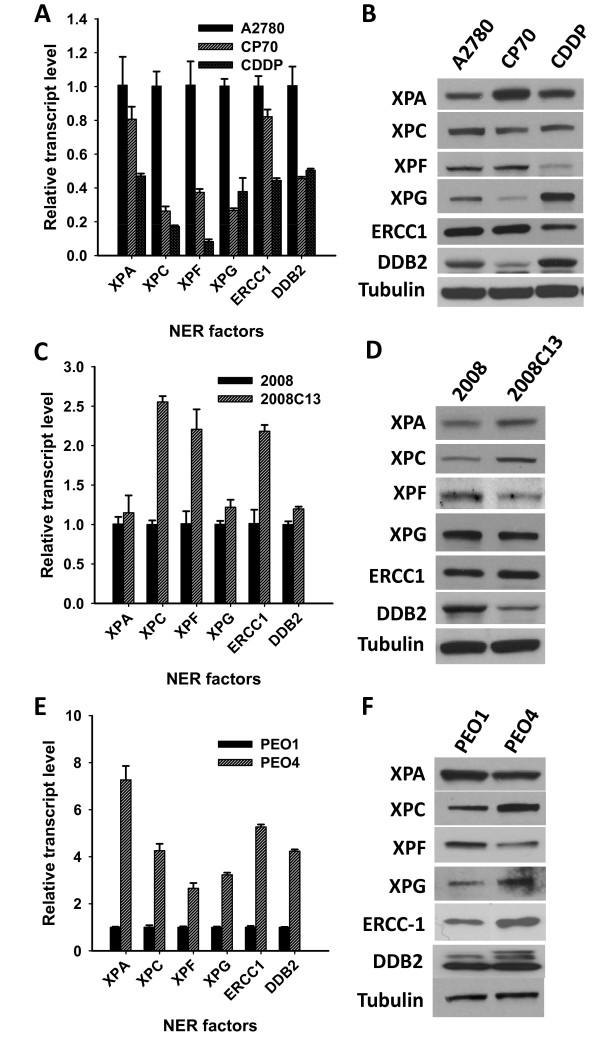
**Expression of various NER related factors at both mRNA and protein levels in multiple cisplatin-sensitive and resistant ovarian cancer cell lines**. Total RNA was isolated and whole cell lysate was prepared from A2780, CP70, and CDDP cells (A, B), 2008 and 2008C13 cells (C, D), PEO1 and PEO4 cells (E, F). RNA was subjected to RT-qPCR to detect the mRNA level of various NER related factors and the relative transcript levels were plotted (A, C, E) (Bar: SD, n = 3). Whole cell lysate was subjected to Western blotting to detect the protein level of these NER factors (B, D, F). Note: Arrow indicates the specific DDB2 band.

The complete processing by NER involves several biochemical steps including damage recognition, dual incision, and gap-filling DNA synthesis [7]. In human cells, the minimal set of NER components involved in the first two steps comprises XPA, RPA, TFIIH, XPC-hHR23B, XPF-ERCC1, and XPG. In order to understand the relationship between the expression of these NER factors and the possibility of developing cisplatin resistance, we evaluated the status of mRNA and protein levels of XPA, XPC, XPF, XPG, ERCC1, and DDB2 in various cisplatin-sensitive and -resistant ovarian cancer cell lines. As shown in Figure 4A, A2780 cell line exhibits higher mRNA levels of all NER factors tested in this study compared with cisplatin-resistant cell lines derived from A2780 cell line. However, the protein expression levels of these factors are not consistent with the mRNA levels. As shown in Figure 4B, in comparison with the parental A2780 cells, CP70 cells have higher protein level of XPA, but lower level of XPC and XPG, while CDDP cells exhibit higher protein level of XPA and XPG, but lower level of XPC, XPF and ERCC1. In addition, 2008 cell line exhibit lower mRNA level for *XPC*, *XPF *and *ERCC1 *compared with its derivative 2008C13 cell line (Figure 4C). However, the protein level of XPF in 2008 cell line is higher than that in 2008C13 (Figure 4D). Furthermore, the mRNA of all NER factors determined in PEO4 cells displayed higher levels than in PEO1 cells (Figure 4E), but the protein levels of XPF and XPA in PEO4 cells are lower than in PEO1 cells (Figure 4F). These data indicate that mRNA level does not always reflect the corresponding protein level of NER factors within cells. Furthermore, the fact that derivative A2780 and 2008 cisplatin-resistant cancer cell lines exhibit normal NER capacity to remove Pt-GG, indicates that low level of certain NER proteins (e.g., XPC and XPG in CP70 cell line, XPC, XPF, and ERCC1 in CDDP cell line, XPF in 2008C13 cell line) may not result in the low NER capacity. Similarly, although PEO4 cells have lower level of XPA and XPF proteins, their NER capacity is higher than PEO1 cells. Thus, the amount of NER factors, by itself, cannot be considered a determinant of NER efficiency and cisplatin sensitivity.

**Figure 5:**
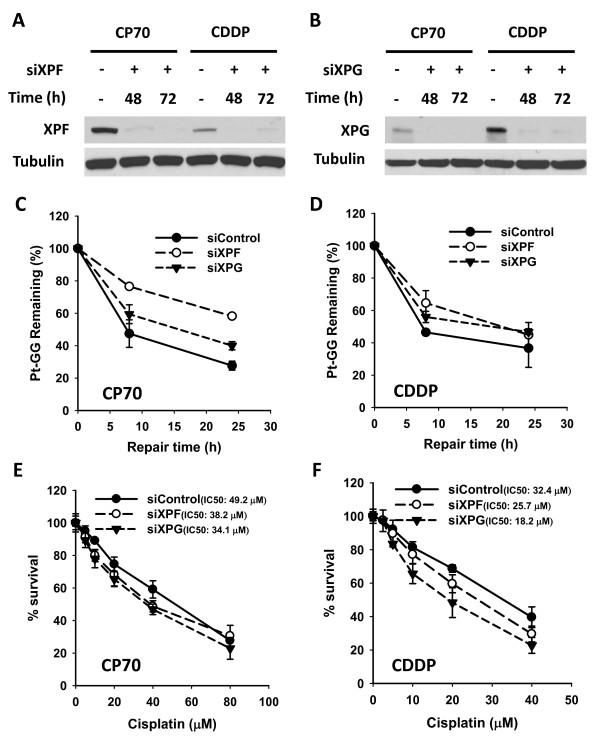
**Effects of XPF or XPG downregulation on the repair of cisplatin-induced intrastrand crosslinks and cisplatin sensitivity in cisplatin-resistant cancer cells**. A, B) CP70 and CDDP cells were transfected with control siRNA, XPF siRNA or XPG siRNA for 48 and 72 h. Whole cell lysates were prepared and subjected to Western blotting for detecting the expression of XPF and XPG. C, D) CP70 and CDDP cells were transfected with siRNA described above for 24 h, pretreated with HU for 24 h, and then treated with cisplatin for 1 h. The cells were then incubated in HU containing medium for 0, 8 and 24 h. The genomic DNA was isolated and analyzed by ISB assay for cisplatin-induced DNA intrastrand crosslinks with anti-Pt-GG antibody. The relative percentage of remaining Pt-GG at different time points is an average of three independent repeats (n = 3, Bar indicates SD). E, F) CP70 and CDDP cells growing in 96-well plates were transfected with above-described siRNA for 24 h. Cells were then treated with cisplatin at the indicated dose for 1 h, and further cultured in drug-free medium for 72 h. Methylene blue stained cells in the treated samples compared to untreated control cells were used to obtain relative cell survival. (n = 4, Bar indicates SD).

To further address the question regarding the contribution of the level of NER factors to NER capacity and cisplatin sensitivity, we knocked down the individual expression of either XPF or XPG in both CP70 and CDDP cells using their corresponding siRNA, and examined their NER capacity to remove Pt-GG and their sensitivity to cisplatin. As shown in Figure 5A &5B, both XPF and XPG siRNA quantitatively knocked down their corresponding target protein expression by greater than 90% in both CP70 and CDDP cell lines. The knockdown of XPG further reduced the NER capacity only slightly in both CP70 and CDDP cells (Figure 5C, D), and sensitized both CP70 and CDDP cell lines to cisplatin, once again by only a slight extent with IC50 of 34.1 μM *vs *49.2 μM in CP70 cells and 18.2 μM *vs *32.4 μM in CDDP cells (Figure 5E, F). In contrast, knockdown of XPF significantly inhibited the NER capacity in both CP70 and CDDP cell lines (Figure 5C, D), while having a lesser influence on their sensitivity to cisplatin, i.e., IC50 of 38.2 μM *vs *49.2 μM in CP70 cells and 25.7 μM *vs *32.4 μM in CDDP cells (Figure 5E, F). This data clearly indicates that the level of NER factors may not have a direct correlation with the sensitivity to cisplatin in ovarian cancer cell lines.

### Downregulation of HRR factor BRCA2, but not NER factor XPA, sensitizes PEO4 cells to cisplatin

**Figure 6:**
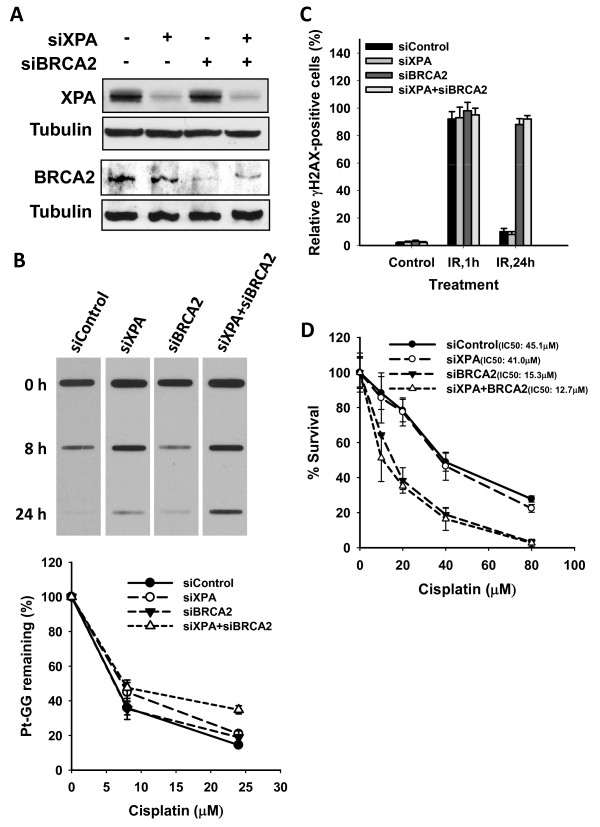
**Targeting HRR is more efficient than targeting NER to sensitize cisplatin-resistant ovarian cancer cells to cisplatin**. A) PEO4 cells were transfected with control siRNA, XPA siRNA, BRCA2 siRNA separately, or both XPA and BRCA2 siRNA for 48 h. Whole cell lysates were prepared and subjected to Western blotting for detecting the expression of XPA and BRCA2. B) PEO4 cells were transfected with various siRNA as described in (A) for 24 h, pretreated with HU for 24 h, and then treated with cisplatin for 1 h. The cells were then incubated in HU containing medium for 0, 8 and 24 h. The genomic DNA was isolated and analyzed by ISB assay for cisplatin-induced 1,2-intrastrand crosslinks with anti-Pt-GG antibody. The relative percentage of remaining Pt-GG at different time points is an average of three independent repeats (n = 3, Bar: SD). C) PEO4 cells growing on coverslips were transfected with various siRNA as described in (A) for 48 h, irradiated with X-ray at 10 Gy, and further cultured for 1 and 24 h. the γH2AX-positive cells were detected with immunofluorescence as described in Materials and Methods. D) PEO4 cells growing in 96-well plates were transfected with various siRNA as described in (A) for 24 h. Cells were then treated with cisplatin at the indicated dose for 1 h, and further cultured in drug-free medium for 72 h. Methylene blue stained cells in the treated samples compared to untreated control cells were used to obtain relative cell survival. (n = 4, Bar: SD).

Downregulation of DNA repair pathways is believed to be one of the useful strategies for sensitizing cancer cells to cisplatin [27]. Since cisplatin-induced intrastrand crosslinks are mainly removed by NER, while the interstrand crosslinks are removed by HRR, we compared the effects of inhibiting NER and HRR pathways on the cisplatin sensitivity of PEO4 cells by knocking down a critical NER factor XPA, or a critical HRR factor BRCA2. Figure 6A showed that siXPA and siBRCA2 can downregulate their corresponding proteins significantly in PEO4 cells. We then analyzed the functional consequence of XPA and BRCA2 knockdown in PEO4 cells. As indicated by Figure 6B, knockdown of XPA, but not BRCA2, compromised the NER capacity, as reflected by more Pt-GG remaining in siXPA transfected cells. In contrast, knockdown of BRCA2, but not XPA, compromised the HRR capacity, as reflected by the persistence of γH2AX up to 24 h following IR treatment in siBRCA2 transfected cells (Figure 6C). As expected, knockdown of XPA and BRCA2 together inhibited both NER and HRR efficiency (Figure 6B,C). Finally, we assessed the sensitivity of PEO4 cells with either XPA knockdown or BRCA2 knockdown to cisplatin. As shown in Figure 6D, siXPA transfection did not enhance PEO4 cells to cisplatin, with an IC50 of 41.0 ± 5.0 μM *vs *45.1 ± 6.6 μM of siControl transfected PEO4 cells. However, siBRCA2 transfection significantly enhanced PEO4 cells to cisplatin, with an IC50 of 15.3 ± 1.3 μM. Double knockdown of XPA and BRCA2 in PEO4 cells conferred the cisplatin sensitivity comparable to BRCA2 knockdown alone, with an IC50 of 12.7 ± 2.6 μM. These data indicated that specifically targeting HRR pathway is more efficient than targeting NER pathway in overcoming cisplatin resistance of ovarian cancers.

## Discussion

### Repair of different cisplatin induced DNA lesions

Cisplatin can induce both intrastrand and interstrand crosslinks in living cells with former accounting for more than 90% of the total DNA damage. Intrastrand crosslinks, the most abundant lesion, can be removed through NER pathway, while interstrand crosslinks are removed through a complex mechanism involving cooperation of several DNA repair pathways, i.e., NER, homologous recombination (HR) and translesion synthesis (TLS) (reviewed in [2]). Since cisplatin-induced DNA damage is the main inducer of apoptosis and contributes to the anti-tumor activity of cisplatin, enhanced DNA repair is believed to be one of the major mechanisms of cisplatin resistance [reviewed in [26]] as it enables tumor cells to overcome cisplatin toxicity. From this standpoint, relationship between DNA repair capacity and cisplatin resistance has been extensively studied [see review [28]], but the basis of contribution of NER to cisplatin resistance remains unsettled. Several groups have compared the NER efficiency between cisplatin-sensitive and -resistant cancer cell lines by detecting the removal rate of cisplatin-induced intrastrand crosslinks *in vivo *or *in vitro*, and suggested that NER efficiency is enhanced in cisplatin-resistant cancer cell lines [29], and an intervention of NER pathway may be utilized for improving the efficacy of cisplatin treatment [29-31]. A recent study, however, raised a concern about the key role of NER pathway in determining cisplatin resistance. There is evidence that a defect in the interaction between ERCC1 and XPA, that disrupts NER, has no major effect on the cellular sensitivity to cisplatin [32]. In addition, Usanova et al [33] have just recently reported that the repair of intrastrand crosslinks is similar in cisplatin sensitive testis tumor cells and resistant bladder cancer cells. Our comprehensive experimentation has now shown that cisplatin-resistant cell lines derived from A2780 and 2008 ovarian carcinoma cell lines do not exhibit any significant enhancement of NER capacity as compared to their parent cell lines and only PEO1-derived cisplatin-resistant ovarian cancer cell line, PEO4, has higher NER capacity than its parental cell line. Considering A2780 and 2008 cell lines possess efficient NER capacity, while PEO1 cell line displays impaired NER function, it seems that enhanced NER only occurs in the cisplatin-resistant cells derived from NER-deficient parental cell lines.

It is worth noting that our ISB assay is highly specific for assessing the removal rate of 1,2-Pt-GG, the most abundant DNA damage induced by cisplatin. Although, the efficiency of removal of 1,2-Pt-GG was comparable between A2780 and CP70/CDDP, or 2008 and 2008C13 cell lines, we cannot rule out the possibility that the removal of other kinds of cisplatin-induced DNA lesions is more efficient in cisplatin-resistant ovarian cancer cell lines than that in sensitive cell lines, which could be contributing to the cisplatin resistance of CP70 cell line [18]. For example, it has been reported that the interstrand crosslinks were removed rapidly from highly cisplatin resistant C80 and C200 cells than their parental sensitive cells [16,34].

### NER factors, repair efficiency, and cisplatin sensitivity

Our thorough evaluation of the NER protein levels (XPA, XPC, XPF, XPG, ERCC1, and DDB2) and corresponding mRNA levels in various cisplatin-sensitive and -resistant ovarian cancer cell lines revealed that the transcript levels of various NER factors do not accurately reflect their protein levels. This is consistent with reports that have shown significantly lower levels of ERCC1, XPF, and XPA protein in testis tumor cell lines do not cohere with the transcriptional efficiency or mRNA stability of the cognate factors [35]. Thus, the transcript level of NER factors detected by quantitative RT-PCR assay, which is widely used in clinical and epidemiological studies, must not be relied as a surrogate of active cellular protein levels and the capacity for DNA repair.

Many studies have demonstrated an increased repair rate in cisplatin-resistant tumor cells [see review [28]]. In addition, overexpression of NER genes has been correlated with repair and resistance [15]. However, our studies demonstrated that ovarian cancer cell lines exhibiting low level of certain NER factors (XPF in CDDP, XPG in CP70) are still resistant to cisplatin, indicating the amount of NER proteins may not be a key determinant of NER efficiency. There are six essential proteins (or complexes) employed in NER, i.e., XPC-hHR23B, TFIIH, XPA, RPA, XPG, and XPF-ERCC1. Lack of any one of these factors can impair NER efficiency *in vivo *and *in vitro *[8]. However, the threshold level of proteins, required for efficient NER, may actually be very low. For example, lower amounts of XPA protein are sufficient to recover UV-resistance of XP-A cells [36]. In the same vein, XPA must be reduced to <10% of the normal wild-type levels to render XPA as a limiting factor for NER and consequently imparting cellular sensitivity [37]. In our study, the very low protein level of XPF in CDDP cells and very low level of XPG protein in CP70 cells did not compromise the NER efficiency of these cells, and emphasize further that the low levels of NER factors are sufficient to execute efficient NER.

Among the basic NER proteins, ERCC1 is one of the most extensively studied NER factors. The *in vitro*, *in vivo*, and clinical studies have demonstrated that high expression of ERCC1 correlates with cisplatin resistance in multiple tumors [38] while knock-down of ERCC1 expression enhances the cisplatin cytotoxicity in cisplatin-resistant ovarian cancer cells [30]. Moreover, patients with completely resected non-small-cell lung cancer and ERCC1-negative tumors appear to benefit from adjuvant cisplatin-based chemotherapy, whereas patients with ERCC1-positive tumors do not [39]. In addition to being involved in NER of intrastrand crosslinks, ERCC1 is also important in the repair of interstrand crosslinks through homologous recombination [40,41]. It is reported that although the excision repair of cisplatin-induced intrastrand crosslinks in PC3 cells was attenuated to the same extent by XPA and ERCC1 knockdown, downregulation of ERCC1, but not XPA, sensitized PC3 prostate cancer cells to cisplatin [42], indicating the role of ERCC1 in the repair of interstrand crosslinks may be more important in cisplatin resistance.

### Targeting DNA repair pathways to improve the efficacy of cisplatin towards resistant cancers

DNA repair proteins can be excellent candidate targets for the development of new therapies to overcome resistance to cancer therapy. Since NER is the major mechanism for removing cisplatin-induced DNA damage, NER factors are being extensively investigated for realizing this goal. However, the effect of down-regulation of NER proteins on cisplatin sensitivity has remained contradictory. For example, it was reported that transfection with antisense XPA RNA could sensitize human lung adenocarcinoma cells to cisplatin [31], whereas, knocking down XPA did not increase cisplatin sensitivity in prostate cancer cell line, although down-regulation of XPA did inhibit cisplatin intrastrand crosslinks repair [42]. In addition, increasing XPA levels in testicular tumor cells by a 10-fold did not increase resistance to cisplatin [43]. In this study, we have found that although the protein levels of XPF and XPG were knocked down more than 90% in cisplatin-resistant CP70 and CDDP cell lines, and XPA was also downregulated significantly in PEO4 cells, the NER efficiency was only inhibited moderately and the sensitivity to cisplatin was increased only slightly. We speculate that despite the very low amount of some NER proteins (not absent) in cancer cells, these levels are sufficient for NER to remove cisplatin-induced intrastrand crosslinks. Thus, reducing NER proteins to similar low levels may not achieve the effective NER inhibition required for increasing cisplatin sensitivity.

As discussed above, cisplatin forms a variety of adducts including DNA intrastrand crosslinks and interstrand crosslinks. Given the cisplatin-induced DNA interstrand crosslinks are mainly repaired by HRR [6], our finding that BRCA2, but not XPA downregulation, enhanced cisplatin sensitivity in PEO4 cells, indicates the minor interstrand crosslinks may be more important than major intrastrand crosslinks to the cell killing, further suggesting that targeting HRR pathway might be more efficient than targeting NER pathway in enhancing the cisplatin sensitivity of ovarian cancer cells [25,32,33].

## Conclusion

Our studies indicate that enhanced NER capacity is not the underlying cause of acquired cisplatin resistance of ovarian cancer cell lines derived from cells that have already high NER efficiency. In contrast, if the cisplatin-sensitive cells have a deficient NER, its derivative resistant cells can exhibit enhanced NER efficiency. Nevertheless, the level of NER factors in ovarian cancer cell lines is neither a determinant of their NER capacity nor of the sensitivity to cisplatin. Interfering with NER pathway through downregulation of NER factors is not an effective means of enhancing cisplatin resistance in ovarian cancer cells. In contrast, the disruption of HR through downregulation of BRCA2 can efficiently sensitize the functional BRCA2-containing cisplatin-resistant ovarian cancer cells to cisplatin treatment. Overall study suggests that the manipulation of HRR but not NER factor expression can serve as an effective strategy for sensitizing cisplatin-resistant tumors to platinating agents.

## Competing interests

The authors declare that they have no competing interests.

## Authors' contributions

KM, CH, Q-EW and JT participated in the Immuno-slot blot and Western blot analysis. CH carried out cell survival analysis and siRNA transfection. Y-WH carried out Real time PCR. Q-EW and AAW conceived, designed, analyzed the data, and prepared manuscript. All authors read and approved the final manuscript.

## References

[B1] Cohen SM Lippard SJ Cisplatin: from DNA damage to cancer chemotherapy Prog Nucleic Acid Res Mol Biol 2001 67 93 130 full_text 11525387 10.1016/s0079-6603(01)67026-0

[B2] Jung Y Lippard SJ Direct cellular responses to platinum-induced DNA damage Chem Rev 2007 107 1387 1407 10.1021/cr068207j 17455916

[B3] Kartalou M Essigmann JM Recognition of cisplatin adducts by cellular proteins Mutat Res 2001 478 1 21 10.1016/S0027-5107(01)00142-7 11406166

[B4] Huang JC Zamble DB Reardon JT Lippard SJ Sancar A HMG-domain proteins specifically inhibit the repair of the major DNA adduct of the anticancer drug cisplatin by human excision nucleasepnas 1994 91 10394 10398 10.1073/pnas.91.22.10394 7937961 PMC45026

[B5] Zamble DB Mu D Reardon JT Sancar A Lippard SJ Repair of cisplatin-DNA adducts by the mammalian excision nuclease Biochemistry 1996 35 10004 10013 10.1021/bi960453+ 8756462

[B6] McHugh PJ Spanswick VJ Hartley JA Repair of DNA interstrand crosslinks: molecular mechanisms and clinical relevance Lancet Oncol 2001 2 483 490 10.1016/S1470-2045(01)00454-5 11905724

[B7] Petit C Sancar A Nucleotide excision repair: From E.coli to man Biochimie 1999 81 15 25 10.1016/S0300-9084(99)80034-0 10214906

[B8] Araujo SJ Tirode F Coin F Pospiech H Syvaoja JE Stucki M Hubscher U Egly JM Wood RD Nucleotide excision repair of DNA with recombinant human proteins: definition of the minimal set of factors, active forms of TFIIH, and modulation by CAK Genes Dev 2000 14 349 359 10673506 PMC316364

[B9] Araujo SJ Nigg EA Wood RD Strong functional interactions of TFIIH with XPC and XPG in human DNA nucleotide excision repair, without a preassembled repairosome Mol Cell Biol 2001 21 2281 2291 10.1128/MCB.21.7.2281-2291.2001 11259578 PMC86862

[B10] Volker M Mone MJ Karmakar P Van Hoffen A Schul W Vermeulen W Hoeijmakers JH van Driel R van Zeeland AA Mullenders LH Sequential assembly of the nucleotide excision repair factors in vivo Mol Cell 2001 8 213 224 10.1016/S1097-2765(01)00281-7 11511374

[B11] Sugasawa K Ng JMY Masutani C Iwai S Van der Spek P Eker A Hanaoka F Bootsma D Hoeijmakers JH Xeroderma pigmentosum group C complex is the initiator of global genome nucleotide excision repair Mol Cell 1998 2 223 232 10.1016/S1097-2765(00)80132-X 9734359

[B12] Moynahan ME Pierce AJ Jasin M BRCA2 is required for homology-directed repair of chromosomal breaks Mol Cell 2001 7 263 272 10.1016/S1097-2765(01)00174-5 11239455

[B13] Sakai W Swisher EM Jacquemont C Chandramohan KV Couch FJ Langdon SP Wurz K Higgins J Villegas E Taniguchi T Functional restoration of BRCA2 protein by secondary BRCA2 mutations in BRCA2-mutated ovarian carcinoma Cancer Res 2009 69 6381 6386 10.1158/0008-5472.CAN-09-1178 19654294 PMC2754824

[B14] Chetrit A Hirsh-Yechezkel G Ben-David Y Lubin F Friedman E Sadetzki S Effect of BRCA1/2 mutations on long-term survival of patients with invasive ovarian cancer: the national Israeli study of ovarian cancer J Clin Oncol 2008 26 20 25 10.1200/JCO.2007.11.6905 18165636

[B15] Saldivar JS Wu X Follen M Gershenson D Nucleotide excision repair pathway review I: implications in ovarian cancer and platinum sensitivity Gynecol Oncol 2007 107 S56 S71 10.1016/j.ygyno.2007.07.043 17884153

[B16] Johnson SW Perez RP Godwin AK Yeung AT Handel LM Ozols RF Hamilton TC Role of platinum-DNA adduct formation and removal in cisplatin resistance in human ovarian cancer cell lines Biochem Pharmacol 1994 47 689 697 10.1016/0006-2952(94)90132-5 8129746

[B17] Masuda H Tanaka T Matsuda H Kusaba I Increased removal of DNA-bound platinum in a human ovarian cancer cell line resistant to cis-diamminedichloroplatinum(II) Cancer Res 1990 50 1863 1866 2306738

[B18] Parker RJ Eastman A Bostick-Bruton F Reed E Acquired cisplatin resistance in human ovarian cancer cells is associated with enhanced repair of cisplatin-DNA lesions and reduced drug accumulation J Clin Invest 1991 87 772 777 10.1172/JCI115080 1999494 PMC329864

[B19] Masuda H Ozols RF Lai GM Fojo A Rothenberg M Hamilton TC Increased DNA repair as a mechanism of acquired resistance to cis-diamminedichloroplatinum(II) in human ovarian cancer cell lines Cancer Res 1988 48 5713 5716 3139281

[B20] Barakat BM Wang QE Han C Milum K Yin DT Zhao Q Wani G Arafa el-SA El-Mahdy MA Wani AA Overexpression of DDB2 enhances the sensitivity of human ovarian cancer cells to cisplatin by augmenting cellular apoptosis Int J Cancer 2010 127 977 988 20013802 10.1002/ijc.25112 PMC4180185

[B21] Koc A Wheeler LJ Mathews CK Merrill GF Hydroxyurea arrests DNA replication by a mechanism that preserves basal dNTP pools J Biol Chem 2004 279 223 230 10.1074/jbc.M303952200 14573610

[B22] Lehmann AR Stevens S A rapid procedure for measurement of DNA repair in human fibroblasts and for complementation analysis of xeroderma pigmentosum cells Mutat Res 1980 69 177 190 10.1016/0027-5107(80)90187-6 6987495

[B23] Liedert B Pluim D Schellens J Thomale J Adduct-specific monoclonal antibodies for the measurement of cisplatin-induced DNA lesions in individual cell nuclei Nucleic Acids Res 2006 34 e47 10.1093/nar/gkl051 16571898 PMC1420801

[B24] Wang QE Zhu Q Wani G El-Mahdy MA Li J Wani AA DNA repair factor XPC is modified by SUMO-1 and ubiquitin following UV irradiation Nucleic Acids Res 2005 33 4023 4034 10.1093/nar/gki684 16030353 PMC1178000

[B25] Sakai W Swisher EM Karlan BY Agarwal MK Higgins J Friedman C Villegas E Jacquemont C Farrugia DJ Couch FJ Urban H Taniguchi T Secondary mutations as a mechanism of cisplatin resistance in BRCA2-mutated cancers Nature 2008 451 1116 1120 10.1038/nature06633 18264087 PMC2577037

[B26] Stewart DJ Mechanisms of resistance to cisplatin and carboplatin Crit Rev Oncol Hematol 2007 63 12 31 10.1016/j.critrevonc.2007.02.001 17336087

[B27] Arora S Kothandapani A Tillison K Kalman-Maltese V Patrick SM Downregulation of XPF-ERCC1 enhances cisplatin efficacy in cancer cells DNA Repair (Amst) 2010 9 745 753 10.1016/j.dnarep.2010.03.010 20418188 PMC4331052

[B28] Martin LP Hamilton TC Schilder RJ Platinum resistance: the role of DNA repair pathways Clin Cancer Res 2008 14 1291 1295 10.1158/1078-0432.CCR-07-2238 18316546

[B29] Ferry KV Hamilton TC Johnson SW Increased nucleotide excision repair in cisplatin-resistant ovarian cancer cells: role of ERCC1-XPF Biochem Pharmacol 2000 60 1305 1313 10.1016/S0006-2952(00)00441-X 11008124

[B30] Selvakumaran M Pisarcik DA Bao R Yeung AT Hamilton TC Enhanced cisplatin cytotoxicity by disturbing the nucleotide excision repair pathway in ovarian cancer cell lines Cancer Res 2003 63 1311 1316 12649192

[B31] Wu X Fan W Xu S Zhou Y Sensitization to the cytotoxicity of cisplatin by transfection with nucleotide excision repair gene xeroderma pigmentosun group A antisense RNA in human lung adenocarcinoma cells Clin Cancer Res 2003 9 5874 5879 14676109

[B32] Orelli B McClendon TB Tsodikov OV Ellenberger T Niedernhofer LJ Scharer OD The XPA-binding domain of ERCC1 is required for nucleotide excision repair but not other DNA repair pathways J Biol Chem 2010 285 3705 3712 10.1074/jbc.M109.067538 19940136 PMC2823511

[B33] Usanova S Piee-Staffa A Sied U Thomale J Schneider A Kaina B Cisplatin sensitivity of testis tumour cells is due to deficiency in interstrand-crosslink repair and low ERCC1-XPF expression Mol Cancer 2010 9 248 10.1186/1476-4598-9-248 20846399 PMC3098011

[B34] Johnson SW Swiggard PA Handel LM Brennan JM Godwin AK Ozols RF Hamilton TC Relationship between platinum-DNA adduct formation and removal and cisplatin cytotoxicity in cisplatin-sensitive and -resistant human ovarian cancer cells Cancer Res 1994 54 5911 5916 7954422

[B35] McGurk CJ Cummings M Koberle B Hartley JA Oliver RT Masters JR Regulation of DNA repair gene expression in human cancer cell lines J Cell Biochem 2006 97 1121 1136 10.1002/jcb.20711 16315315

[B36] Muotri AR Marchetto MC Suzuki MF Okazaki K Lotfi CF Brumatti G Amarante-Mendes GP Menck C Low amounts of the DNA repair XPA protein are sufficient to recover UV-resistance Carcinogenesis 2002 23 1039 1046 10.1093/carcin/23.6.1039 12082027

[B37] Koberle B Roginskaya V Wood RD XPA protein as a limiting factor for nucleotide excision repair and UV sensitivity in human cells DNA Repair (Amst) 2006 5 641 648 10.1016/j.dnarep.2005.12.001 16413230

[B38] Altaha R Liang X Yu JJ Reed E Excision repair cross complementing-group 1: gene expression and platinum resistance Int J Mol Med 2004 14 959 970 15547660

[B39] Olaussen KA Dunant A Fouret P Brambilla E Andre F Haddad V Taranchon E Filipits M Pirker R Popper HH Stahel R Sabatier L Pignon JP Tursz T Le Chevalier T Soria JC DNA repair by ERCC1 in non-small-cell lung cancer and cisplatin-based adjuvant chemotherapy N Engl J Med 2006 355 983 991 10.1056/NEJMoa060570 16957145

[B40] Ahmad A Robinson AR Duensing A van Drunen E Beverloo HB Weisberg DB Hasty P Hoeijmkers JH Niedernhofer LJ ERCC1-XPF endonuclease facilitates DNA double-strand break repair Mol Cell Biol 2008 28 5082 5092 10.1128/MCB.00293-08 18541667 PMC2519706

[B41] Niedernhofer LJ Odijk H Budzowska M van Drunen E Maas A Theil AF de Wit J Jaspers NG Beverloo HB Hoeijmakers JH Kanaar R The structure-specific endonuclease Ercc1-Xpf is required to resolve DNA interstrand cross-link-induced double-strand breaks Mol Cell Biol 2004 24 5776 5787 10.1128/MCB.24.13.5776-5787.2004 15199134 PMC480908

[B42] Cummings M Higginbottom K McGurk CJ Wong OG Koberle B Oliver RT Masters JR XPA versus ERCC1 as chemosensitising agents to cisplatin and mitomycin C in prostate cancer cells: role of ERCC1 in homologous recombination repair Biochem Pharmacol 2006 72 166 175 10.1016/j.bcp.2006.04.025 16756962

[B43] Koberle B Roginskaya V Zima KS Masters JR Wood RD Elevation of XPA protein level in testis tumor cells without increasing resistance to cisplatin or UV radiation Mol Carcinog 2008 47 580 586 10.1002/mc.20418 18240296

